# Nucleic acid chemistry

**DOI:** 10.3762/bjoc.10.311

**Published:** 2014-12-10

**Authors:** Hans-Achim Wagenknecht

**Affiliations:** 1 Institute of Organic Chemistry, Karlsruhe Institute of Technology (KIT), Fritz-Haber-Weg 6, 76131 Karlsruhe, Germany

**Keywords:** nucleic acids

Since the discovery of the structure of the DNA double helix in 1953 by Watson and Crick [1], we know that DNA is of critical importance, carrying the genetic information for all living organisms. Only a few years later appeared the first reports on the chemical synthesis of oligonucleotides with a natural 3'-5' phosphodiester linker by Michelsen and Todd [2]. It took another two decades until the first step towards building block chemistry (the so-called phosphoramidte chemistry) was published by Letsinger and Lunsford, enabling efficient and fully synthetic access to oligonucleotides [3]. These authors discovered that DNA building blocks based on phosphorous(III) were significantly more reactive than phosphordiesters or -triesters. Finally, this approach using phosphoramidites as nucleoside building blocks was significantly further developed in 1981 by Beaucage and Caruthers [4]. Since then, oligonucleotides of up to 50-mers in length have become available by an extremely efficient solid-phase methodology that runs automatically on machines, so-called DNA synthesizers. With special care during each synthesis step, even longer oligonucleotides can be prepared in a similar, almost routine fashion. The field of nucleic acid chemistry has exploded: natural and artificial functionalities, as well as probes, markers or other biologically active molecules, can be synthetically introduced into DNA (as well as RNA) by preparing the corresponding artificial DNA and RNA building blocks. More recently, the chemistry was further developed for building blocks that are otherwise synthetically not obtainable. In such cases, postsynthetic strategies can allow for the desired oligonucleotides modification. This becomes an even more important issue for functionalities, probes or biologically relevant molecules that are incompatible with routine phosphoramidite chemistry. Although nucleic acid chemistry appears to be a mature part of organic and bioorganic chemistry, the many questions that are still being raised by research in biology and chemical biology give sufficient motivation to continue to synthesize new nucleic acid probes and thereby further develop nucleic acid chemistry. Moreover, in addition to their biological functionality, DNA and RNA are considered as increasingly important architectures and scaffolds for two- and three-dimensional objects, networks and materials for nanosciences. In conclusion, nucleic acid chemistry continues to maintain its appeal and affords good reason to focus on in this Thematic Series of the Beilstein Journal of Organic Chemistry.

Hans-Achim Wagenknecht

Karlsruhe, November 2014
